# The Influence of Tone Inventory on ERP without Focal Attention: A Cross-Language Study

**DOI:** 10.1155/2014/961563

**Published:** 2014-08-28

**Authors:** Hong-Ying Zheng, Gang Peng, Jian-Yong Chen, Caicai Zhang, James W. Minett, William S-Y. Wang

**Affiliations:** ^1^School of Software Engineering, Shenzhen Institute of Information Technology, Shenzhen 518172, China; ^2^Department of Electronic Engineering, The Chinese University of Hong Kong, Shatin, Hong Kong; ^3^CUHK-PKU-UST Joint Research Centre for Language and Human Complexity, The Chinese University of Hong Kong, Hong Kong; ^4^Department of Linguistics and Modern Languages, The Chinese University of Hong Kong, Hong Kong; ^5^Shenzhen Institutes of Advanced Technology, Chinese Academy of Sciences, Shenzhen 518055, China; ^6^Department of Computer Science and Technology, Shenzhen University, Shenzhen 518060, China

## Abstract

This study investigates the effect of tone inventories on brain activities underlying pitch without focal attention. We find that the electrophysiological responses to across-category stimuli are larger than those to within-category stimuli when the pitch contours are superimposed on nonspeech stimuli; however, there is no electrophysiological response difference associated with category status in speech stimuli. Moreover, this category effect in nonspeech stimuli is stronger for Cantonese speakers. Results of previous and present studies lead us to conclude that brain activities to the same native lexical tone contrasts are modulated by speakers' language experiences not only in active phonological processing but also in automatic feature detection without focal attention. In contrast to the condition with focal attention, where phonological processing is stronger for speech stimuli, the feature detection (pitch contours in this study) without focal attention as shaped by language background is superior in relatively regular stimuli, that is, the nonspeech stimuli. The results suggest that Cantonese listeners outperform Mandarin listeners in automatic detection of pitch features because of the denser Cantonese tone system.

## 1. Introduction

Pitch perception is very important for tone languages, which utilize pitch patterns to distinguish lexical meanings. For example, in Mandarin, a tone language, the same segmental syllable /ma/ means “mother” when produced with a high level pitch contour but means “hemp” when produced with a high rising pitch contour [1]. Tone languages usually have different tone inventories. For example, Mandarin has four lexical tones; Cantonese, another tone language, has six lexical tones. The language backgrounds not only influence the perception of pitches in speech [2, 3] but also generalize to nonspeech processing under certain stimulus and task conditions [3, 4]. Recently, many efforts have been devoted to studying the neural bases of language influence on pitch perception under various conditions regarding the types of stimulus and task. Electrophysiological signals, which can reflect neural activity involved in cognitive processing at various levels, are widely used to explore how the brain processes language, for example, at long-term memory traces level [5], at semantic memory level [6], and at syntactic grammatical level [7], and so forth.

Before the pitch signal is transmitted to the cortex level, the frequency following responses (FFR) of pitch at the brainstem have been shown to be sensitive to language-relevant aspects of pitch contours but not specific to speech [8]. To record FFR at the brainstem, subjects do not need to pay attention to the stimuli. Moreover, the information of lexical item has not been fully retrieved. Therefore, brainstem responses to pitch contours carried by speech sound do not differ from those carried by nonspeech sound [9]. On the other hand, when subjects pay attention to the incoming stimuli, the information of lexical item gets fully processed. Therefore, pitch perception differs in speech and nonspeech [8]. For example, brain imaging data have pinpointed distinct brain regions in response to pitch contours superimposed on linguistic (pseudoword) and nonlinguistic (hum) carriers [10]. This brain imaging finding is consistent with behavioural results [11], which show selective enhancement of pitch discrimination in speech context for native tone language speakers. This enhancement may result from the phonological processing, which can be indexed by a late positive event-related potential (ERP) component, P300, which is usually recorded with focal attention [12, 13].

The phonological processing of pitch contours was investigated in the study of [13] through a 2 × 2 × 2 design on category status (within versus across-category), context type (speech versus nonspeech), and the tone inventories (Mandarin versus Cantonese). In the study, the enhancement of P300 amplitude from across-category stimuli is more obvious in speech stimuli than in nonspeech stimuli. More interestingly, this selective enhancement of pitch discrimination in speech context is statistically significant by Cantonese speakers but not by Mandarin speakers. The authors attribute this finding to the influence of different tone inventories in the two language systems. Mandarin tones tend to be produced distinctly from each other, allowing the Mandarin speakers to discriminate them readily. On the other hand, the tone system of Cantonese is acoustically denser than that of Mandarin and there is significant overlap in pitch height and slope for the Cantonese tones. The denser tone system might require the Cantonese speakers to make finer distinctions in pitch height and slope in order to discriminate certain tones than the Mandarin speakers.

Although the study compared different conditions involving the category status and context type, it did not compare different levels of attention. Therefore, it is not clear whether the observed group difference stems from the explicit category information (lexical items distinguished by pitch contours) only, which often requires focal attention to do online judgment, or also from automatic feature detection which can be done even without focal attention. In the oddball paradigm, the brain must form a representation of the repeated auditory stimulus before the occurrence of a deviant stimulus, regardless of attention [14]. However, depending on the status (absence or presence) of subjects' focal attention, the oddball paradigm will elicit different ERP components, indexing distinctive stages of brain processing. P300, which may index the phonological processing, often is elicited with subjects' focal attention. On the other hand, other ERP components (e.g., MMN, mismatch component [15]), which are elicited without subject's attention, may index the automatic feature detection.

In the present experiment, we examine native Mandarin and Cantonese subjects' electrophysiological responses to the same set of speech and nonspeech tonal stimuli as those used in [13]. Subjects are instructed to ignore these stimuli presented in the oddball paradigm. Specifically, three questions will be investigated: (1) whether or not the automatic detection of across-category deviant is easier than that of within-category deviant, (2) whether or not the difference in brain responses which exists between two types of deviants differs in speech and nonspeech contexts, and (3) whether or not Cantonese and Mandarin speakers perform differently on the same set of stimuli.

## 2. Materials and Methods

### 2.1. Participants

Fifteen native Mandarin speakers (7 F; age: 22.7 ± 2.2) and fifteen native Hong Kong Cantonese speakers (7 F; age: 21.6 ± 2.2), with normal hearing and no reported history of neurological illness, were paid to participate in the experiment. All subjects were right-handed university students. Before the age of seven, no subject of either group had learned the first language of the other group or received musical training. Approval to conduct the experiment was obtained from the Survey and Behavioural Research Ethics Committee of the Chinese University of Hong Kong.

### 2.2. Stimuli

This study includes two sets of stimuli, speech syllable /i/ and nonspeech complex tone. Each set included three stimuli drawn from a continuum of eleven stimuli, that is, a within-category deviant (stimulus number 1), a standard (number 4), and an across-category deviant (number 7).

**Figure 1:**
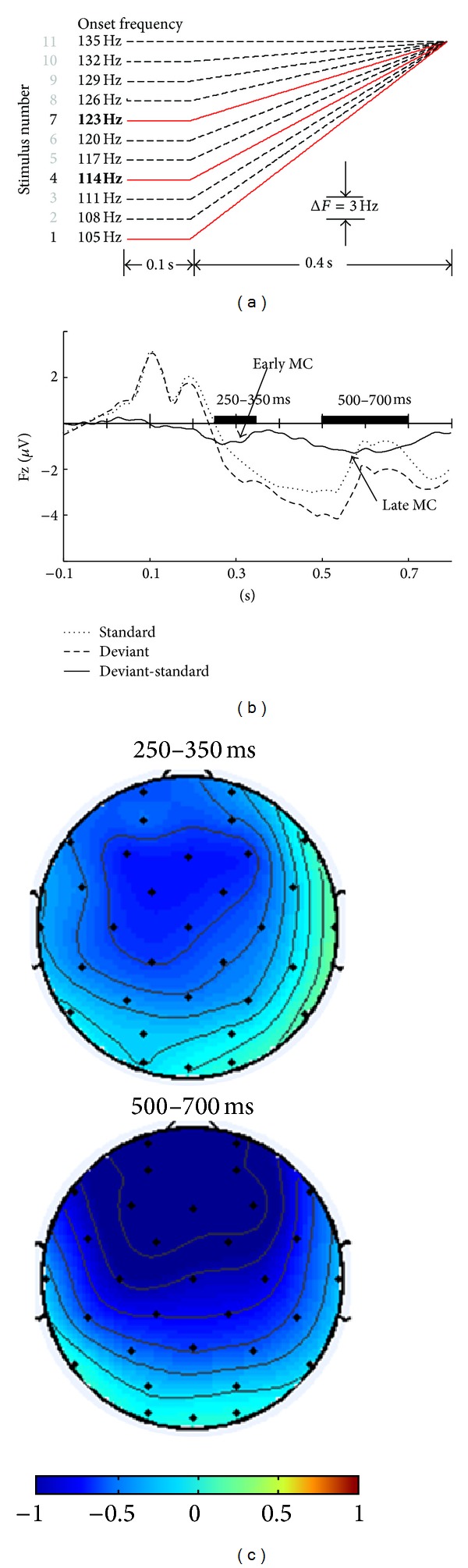
Pitch contours of stimuli, number 1 within-category deviant, number 4 standard, and number 7 across-category deviant (a). ERPs for standard (averaged over two language groups and two types of context), deviants (averaged over two language groups, two types of context, and two types of deviants), and the difference wave between deviants and standard on Fz (b). Topographic distribution of early mismatch component (250–350 ms) and late mismatch component (500–700 ms) (c).

The eleven speech stimuli, each of duration 500 ms, were synthesized from the Tone 1 syllable /i/ uttered by a native Mandarin speaker with the pitch contours manipulated as illustrated in Figure 1(a). End points of this pitch continuum formed bilinear approximations of the high-level (Tone 1) and rising tones (Tone 2) in both Mandarin and Cantonese. The category boundary was determined based on the identification test, and the naturalness rating for the synthesized stimuli obtained from Mandarin and Cantonese speakers was comparable [3, 13]. Eleven additional nonspeech stimuli were synthesized from a complex tone (saw wave) with the same pitch contours as the speech stimuli. Loudness of the two sets of stimuli was comparable and the intensity envelopes of the two stimulus sets were closely matched.

### 2.3. Procedure

The stimuli (Figure 1(a)) were presented in an oddball paradigm. 1200 trials of each stimulus set (80% standards and 10% for each type of deviant; 500 ms interstimulus interval, ISI) were pseudorandomized, with at least two standards preceding each deviant. Two stimulus sets (i.e., speech and nonspeech) were presented in counterbalanced order. All stimuli were presented binaurally to subjects via a pair of E*·*A*·*RTone 3A insert earphones. Subjects sat in an acoustically shielded booth and were instructed to watch a self-selected muted movie with subtitles while ignoring the sound stimuli. Throughout the experiment, electroencephalographic data were recorded using a 32-channel ActiveTwo Biosemi EEG system. Fp1, Fp2, and two additional channels attached near the outer canthus of each eye were used to monitor artifacts due to eye activities. Moreover, two additional channels attached at each mastoid were used as references. The recordings were digitized at a rate of 1024 Hz.

After the EEG recordings, a behavioural same/different* discrimination* posttest with the stimulus pairs 1–4, 4–1, 4–7, and 7–4 in both contexts was conducted to confirm the categorical status of these stimuli. In this task, subjects were instructed to discriminate whether a pair of stimuli (500 ms ISI) were the same or not by pressing one of the two buttons within 3 s. Seven repetitions of each pair were presented to subjects in separate blocks. Results from one extra practice block were excluded from the analysis.

### 2.4. Data Analysis

Each comparison unit was comprised of all trials in four types of comparisons (AB, BA, AA, and BB). Discrimination response (*D* hereafter) for each comparison unit is defined by percentage of correct responses from both the same and the different pairs (see also [16, 17]).

The EEG recordings were re-referenced offline against average-mastoid, and 0.5–30 Hz band-pass was filtered. ERPs were 900 ms in duration with a 100 ms prestimulus baseline obtained from each condition and each subject. Trials with ocular artifacts were excluded from averaging. Mismatch components (MC) were obtained by subtracting the ERP of the standard from that of each type of deviant. Two negative components were determined from the maximal negativity of the grand-averaged difference waves across all experimental conditions (see Figure 1(b)). Eight frontal central electrodes—F3, Fz, F4, C3, Cz, C4, FC1, and FC2—were selected according to the region of interest and confirmed by the topographic distribution maps (see Figure 1(c)). The ERPs of temporal electrodes (FC5, FC6, T7, and T8) were too weak to get any significant effect. Therefore, no further analyses were applied on them. The early MC and late MC, with width of 60 ms and 100 ms, were centred on the individual peak within the negative deflection window judged from the grand-averaged difference wave at 200–350 ms and 400–700 ms, respectively.

Three-way mixed design repeated-measures analysis of variance (MANOVA) was carried out on the behavioural and electrophysiological responses. Two within-subject factors were *context* (speech versus nonspeech) and* category* (across-category versus within-category). One between-subjects factor was* language* (Mandarin versus Cantonese). The dependent variable in the behavioural responses was* D*, while the dependent variables in ERP were mean amplitude and peak latency from the early and late MC, respectively.

The *P* values of the post hoc *t*-tests were all corrected for multiple comparisons wherever appropriate. All tests of significance were conducted at *P* < 0.05 after the correction.

## 3. Results

### 3.1. Behavioural Data

**Figure 2:**
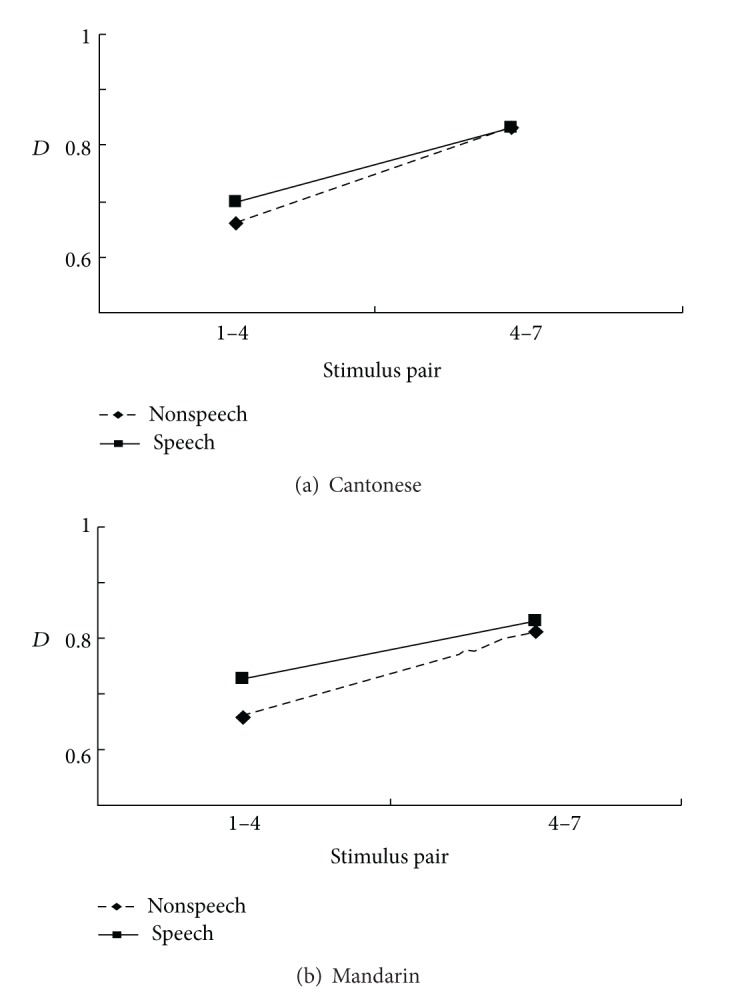
Discrimination response *D* for Cantonese (a) and Mandarin (b).

There was only a significant main effect of* category* (Figure 2),* F*(1, 28) = 45.557, *P* < 0.001, which indicated that the discrimination of pair 4–7 (*D* = 0.828 ± 0.013) was easier than that of pair 1–4 (*D* = 0.686 ± 0.018).

### 3.2. Electrophysiological Data

#### 3.2.1. Early Mismatch Component

**Figure 3:**
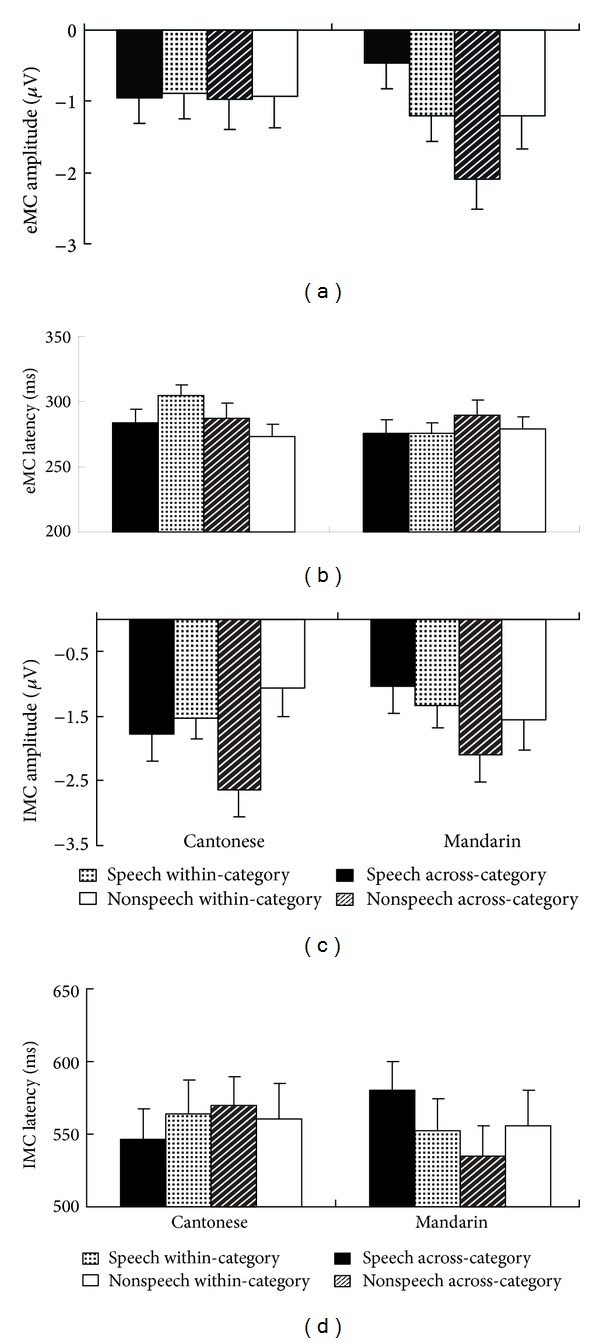
Mismatch component (MC) averaged over eight electrodes from Cantonese and Mandarin participants. The mean amplitude of early MC (eMC) (a), the peak latency of early MC (b), the mean amplitude of late MC (c), and the peak latency of late MC (lMC) (d).

The MANOVA did not reveal any significant effect of mean amplitude (Figure 3(a)); but there was a signification* category* ×* context* interaction effect of the peak latency (Figure 3(b)),* F*(1, 28) = 4.551, *P* < 0.05. No other effects reached significance. The interaction effect on peak latency indicated that across-category and within-category deviants were detected at with different levels of difficulties (as reviewed in [15]), which depended on the context (carrier).

#### 3.2.2. Late Mismatch Component

There was not any significant effect on peak latency (Figure 3(d)), but only a signification* category* ×* context* interaction effect of mean amplitude (Figure 3(c))* F*(1, 28) = 6.803, *P* < 0.05. Moreover, post hoc analyses revealed a significant* category* effect—larger electrophysiological responses from across-category deviants than within-category deviants were elicited from Cantonese speakers for nonspeech stimuli* F*(1, 14) = 6.87, *P* < 0.05.

## 4. Discussion

### 4.1. Behavioural Data

The posttest behavioural task shows that discrimination of the across-category pair is easier than that of the within-category pair, regardless the type of context or language background. This result verifies that the selection of across-category deviant (number 7) and within-category deviant (number 1) relative to the standard stimulus (number 4) in the electrophysiological recording is appropriate.

### 4.2. Early Mismatch Component

The early MC showed an interaction effect of peak latency between category status and context type. It may reflect that across-category and within-category deviants are detected with different levels of difficulties (as reviewed in [15]), which further depends on the context (carrier). However, since no other effects are obtained in the post hoc analyses, it is not discussed in detail here. In contrast to other studies [18, 19], the present study does not obtain a main category effect in the early time window without focal attention. The absence of a main category effect may be due to the usage of a much smaller physical distance (9 Hz) than those in other studies [18, 19], which often used a much larger distance (above 30 Hz) between the deviants and the standard. Although in active processing, the distance of 9 Hz is well enough to elicit categorical effect due to the facilitation of explicit category information, such a small distance may not elicit a robust category effect at this early time window in passive processing. It would be worthy investigating the relationship of just noticeable discrimination distance in categorical perception between with and without focal attention in future investigation.

### 4.3. Late Mismatch Component

#### 4.3.1. Category Effect

At the late time window, across-category deviant elicits a larger MC than within-category deviant in nonspeech context only. This suggests that the category effect for tones may be present even without focal attention, although at a much later time than the classic MMN [15]. The absence of category effect in speech context may be due to different spectral structures between speech and nonspeech contexts (as discussed in [16]). Tone perception mainly relies on perception of pitch contours, whose information can be obtained from the harmonic structure. The harmonic structure of the nonspeech context is simpler and more regular than that of the speech context. Therefore, the category effect may be stronger in the nonspeech context without focal attention in the present study. Another possible explanation for the absence of category effect in speech context is that the activation of auditory cortex, the neural generator of mismatch component, is suppressed by the perception of visual stimuli, the movie as well as its subtitles [20]. Moreover, a study has reported that neural activity in the temporal region is decreased while subjects attended to both visual and auditory stimuli [21]. Although subjects attended to the subtitles in both speech and nonspeech conditions, it is likely that the linguistic interference from subtitles is greater for speech context than for nonspeech context.

The late MC has been reported to reflect the summation of MMN generators and memory trace formation on gestalt bases [22] and is observed in response to changes in unattended speech or nonspeech stimuli [23], from new born infants [23, 24], children [22, 23], and adults [25]. It has been suggested that the late MC, like the classic MMN, is a prominent tool in studying speech perception and learning [23]. This late MC is probably not linked with either sensory or attentional processing of sound differences but reflects higher-order, cognitive, albeit not explicitly conscious processing of sound differences [26]. However, the late negativity has not always been found and studied in all passive oddball studies, partly because the ISI was too short to elicit the component in some earlier studies (e.g., [23]). In the present study, where the physical distance between the deviants and the standard is small, the late MC may be a more reliable indicator for the category effect, which merits more studies to further investigate the function of late MC.

#### 4.3.2. Language Effect

The post hoc tests reveal that the category effect from nonspeech context is only present in the electrophysiological responses of Cantonese speakers. No effect reaches significance in the responses of Mandarin speakers. This result is consistent with the hypothesis proposed in the previous study [13] that Cantonese speakers have to make finer distinctions in the perception of pitch height and slope than Mandarin speakers in order to discriminate the more densely distributed tones. A recent study which compares the ERP correlates of auditory pitch feedback between Mandarin and Cantonese speakers also suggests that Cantonese speakers may require more highly tuned perceptual abilities for tone discrimination than Mandarin speakers due to their denser tone inventories than Mandarin [27].

#### 4.3.3. Context Effect

There is no evidence for the category effect in the speech context without focal attention in the present study. In contrast, with focal attention, category effect is stronger in the speech context than in the nonspeech context [13]. At the subcortex level where the auditory signal has not been transmitted to the cortex yet, there is no context effect for pitch perception [9]. At the cortex level where the phonological processing with focal attention takes place, the context effect indexed by P300 reaches significance [13]. For the intermediate stage, that is, when the auditory signal reaches the cortex level but without focal attention, the context effect remains controversial ([18, 28] versus [29]). Using the same experimental paradigm and stimuli, the results from the present study complement the early findings [13] by investigating the pitch perception without focal attention. The present study suggests that Cantonese speakers outperform Mandarin speakers in automatic feature detection. Such better performance in feature detection likely contributes to the P300 CP effect for Cantonese speakers. Therefore, the better phonological processing ability may not be the sole reason to explain the P300 CP effect in [13]. Nonetheless, the result obtained from the present study, which demonstrates greater CP effect for Cantonese speakers than for Mandarin speakers even without focal attention, is also likely due to the denser Cantonese tonal inventory.
